# Synergy of Low Injection Resistance and High Plugging in a High-Strength Nanobentonite/Amphoteric Polymer Gel

**DOI:** 10.3390/gels11110847

**Published:** 2025-10-23

**Authors:** Huaizhu Liu, Guiqiang Fei, Kaiping Tian, Zhao Zhu, Donghang Ji, Houyong Luo

**Affiliations:** 1 Department of Chemistry and Chemical Engineering, Shaanxi University of Science and Technology, Xi’an 710021, China; zcy_liuhzh@petrochina.com.cn (H.L.); zhuzhao@sust.edu.cn (Z.Z.); jidonghang@sust.edu.cn (D.J.); 2 Tangshan Jiyou Ruifeng Chemical Limited Company, Tangshan 063500, China; 3 Xi’an Key Laboratory of Auxiliary Chemistry and Technology for Oil and Gas Fields, Shaanxi University of Science and Technology, Xi’an 710021, China

**Keywords:** low-permeability reservoir, injectability, plugging strength, plugging system, enhanced oil recovery

## Abstract

Long-term water flooding development has exacerbated reservoir heterogeneity, and traditional polymer gels are unable to simultaneously meet the requirements of high injectability and strong plugging strength. If the viscosity of the polymer is high, its injectability will be poor; on the contrary the viscosity is low, the plugging strength will be poor, which severely restricts the oil recovery effect. This study synthesized an NBAP through free radical polymerization followed by a substitution reaction, and a plugging system (NBAP-B1) was subsequently formed by combining the polymer with a Cr^3+^ crosslinking agent. Rheological experiments demonstrated that the system exhibited significant shear thinning behavior, as well as excellent temperature and salt resistance. Gelation experiments indicated that the NBAP-B1 system featured controllable gelation time (20~150 h) and high gelation strength (J grade), along with excellent resistance to both high temperature and high salinity. Microscopic analysis revealed that the gel formed by NBAP-B1 possessed a dense and uniform three-dimensional network structure. Injection and plugging experiments demonstrated that NBAP-B1 exhibited optimal injectability and outstanding plugging performance. Additionally, profile control and displacement tests revealed a 18.37% enhancement in oil recovery efficiency by water flooding after the application of NBAP-B1 for conformance control. Collectively, these results demonstrate that the NBAP exhibits significantly superior performance compared to single component systems. It combines excellent injectability with high strength plugging capability, offering an effective approach for enhancing oil recovery in low permeability reservoirs.

## 1. Introduction

Petroleum plays an indispensable and foundational role in areas such as energy supply, transportation, and chemical production, exerting a profound influence on society economic development [1,2,3]. However, amid continuously growing global energy demand, the petroleum supply is facing significant challenges, with low extraction efficiency emerging as a critical bottleneck constraining the development of the petroleum industry [4,5,6]. Research indicates that low-permeability reservoirs already account for more than 70% of newly developed oil fields globally, yet their average recovery rate remains below 30%, resulting in significant resource wastage [7,8]. As a primary stimulation technique for enhancing production in such reservoirs, fracturing technology aims to improve water-flooding efficiency by increasing reservoir permeability [9,10,11,12,13]. However, long-term fracturing operations often lead to the formation of preferential flow channels, which exacerbate reservoir heterogeneity and cause issues such as water channeling and ineffective recycling of injected water [14,15,16]. Taking the Gudao Block of Shengli Oilfield as an example, during the ultrahigh water cut development stage, the comprehensive water cut can exceed 90%, while the oil recovery rate remains below 10%. Notably, the proportion of high water-cut wells ranges from 40% to 60%. Therefore, addressing the issue of high water cut has become a critical scientific challenge that must be resolved to enhance oil recovery in low-permeability reservoirs [17,18,19,20,21].

As an effective method for addressing high water cut in oil reservoirs, profile control and water shutoff technology relies on the injection of chemical agents to selectively block high-permeability channels, alter fluid flow paths, and increase sweep efficiency, thereby displacing remaining oil from low-permeability zones [22,23]. Conventional profile control and water shutoff agents commonly in use include gels, gelants, microspheres, and resins, among other traditional materials [24,25,26]. However, as reservoir development conditions become increasingly complex, the application of these conventional agents in low-permeability reservoirs (permeability < 10 mD) faces significant technical challenges. Specifically, although high-viscosity plugging systems can form high-strength gel blockages, their injection becomes difficult in low-permeability reservoirs due to the fine pore-throat structures, significantly limiting the conformance volume [27,28,29,30]. On the other hand, low-viscosity systems offer favorable injectivity but often fail to achieve effective plugging due to insufficient gelation strength [30,31,32,33]. This inherent contradiction between injectivity and plugging strength significantly restricts the effectiveness of conventional plugging agents in low-permeability reservoirs [34,35,36,37].

In recent years, numerous studies have sought to enhance plugging agent performance through two main approaches, one of which involves the development of composite material systems. For instance, Song et al. developed P(AA-AM-SA)/TiO_2_ nanocomposite microspheres, which demonstrated exceptional plugging performance (plugging rate > 95.3%) under high-temperature and high-salinity conditions, resulting in a 12.89% increase in oil recovery [38]. The incorporation of nanomaterials has significantly enhanced the mechanical strength of polymers; however, the multi-component nature of these systems often leads to high costs and operational complexity [39,40,41,42]. Another approach involves chemical modification. For example, Pu et al. [43] synthesized an amphoteric polyacrylamide (LHPAM), which exhibits notably low viscosity and favorable injectivity. After crosslinking with Cr^3+^, the system achieved a plugging efficiency exceeding 90%. However, such gels often fail to maintain long-term stability under high-temperature and high-salinity conditions. To address this limitation, this study proposes a novel nanocomposite plugging agent designed to simultaneously enhance injectivity and plugging strength. The approach involves incorporating both anionic and cationic functional groups into the polymer backbone. This dual functionalization induces polymer chain coiling through ionic interactions, resulting in low viscosity and excellent injectivity; Meanwhile, bentonite nanoparticles are anchored to the polymer backbone via chemical bonding, significantly enhancing the mechanical strength of the three-dimensional network within the polymer structure. This unique molecular design leverages the synergistic effect of nanomaterial reinforcement and zwitterionic modification, successfully addressing the long-standing challenge of achieving both low viscosity and high mechanical strength in conventional plugging agents for low-permeability reservoirs. To date, there have been limited reports on the use of low-viscosity yet high-strength polymer gels for profile control and water shutoff in such reservoirs to enhance oil recovery. The proposed method demonstrates significant potential for application in the development of unconventional reservoirs.

Building on this foundation, the objective of this study is to develop a low-viscosity, high-strength nanopolymer gel system to effectively enhance oil displacement efficiency in low-permeability reservoirs. Using acrylamide (AM) as the monomer, the polymer was functionalized via free-radical polymerization and substitution reactions to introduce 3-chloro-2-hydroxypropyltrimethylammonium chloride (cationic group) and sodium 2-chloroethylsulfonate (anionic group). Bentonite nanoparticles were subsequently incorporated, resulting in a nanocomposite termed NBAP (Nanocomposite Bentonite/Amphoteric Polyacrylamide). and was further formulated with polyvinyl alcohol and Cr^3+^ crosslinker to form a nanopolymer plugging system designated as NBAP-B1. The structure of the material was characterized using Fourier transform infrared spectroscopy (FTIR) and scanning electron microscopy (SEM). Its rheological properties, gelation behavior, injectivity, and plugging performance were evaluated through rheological tests, gelation experiments, and physical simulation studies. These analyses elucidate the synergistic effect of low injection resistance and high plugging efficiency in NBAP-B1 and reveal its mechanism for profile control and oil displacement in low-permeability reservoirs. The findings of this study provide important theoretical insights and technical support for the efficient development of low-permeability reservoirs, offering significant potential for enhancing oil recovery.

## 2. Results and Discussion

### 2.1. Structural Characterization

#### 2.1.1. FTIR

**Figure 1:**
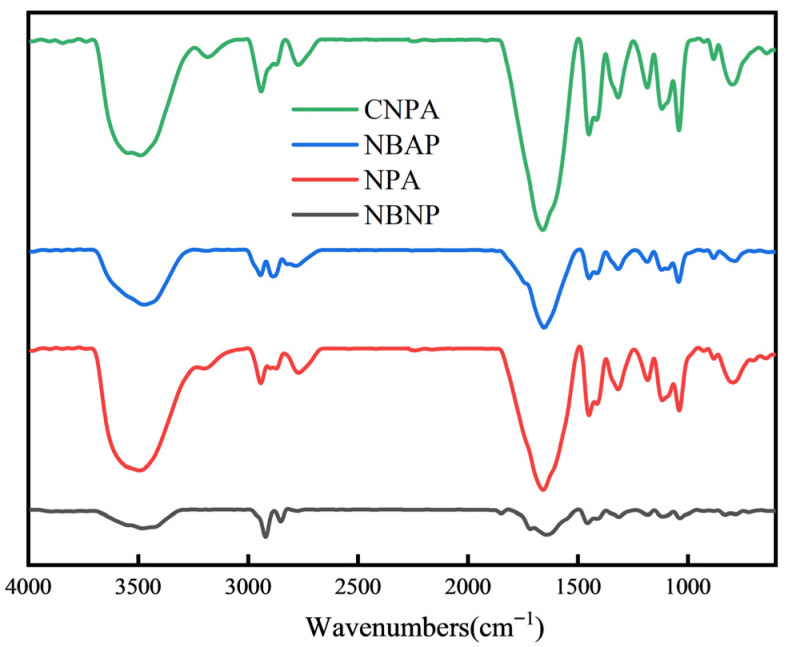
FTIR spectra of the different samples.

Figure 1 presented the Fourier transform infrared (FTIR) spectroscopy results of the different samples. A broad absorption band was observed in the range of 3400–3500 cm^−1^, which is primarily attributed to the stretched vibration of the NH_3_^+^ group within the polymer molecules. It is noteworthy that the hydroxyl group (OH) in 3-chloro-2-hydroxypropyltrimethylammonium chloride present in both CNPA and NBAP may also contribute to the spectral features observed in this region. The characteristic peak at 2920 cm^−1^ corresponds to the symmetric stretched vibration of methylene groups (-CH_2_-) on the polymer backbone. Notably, CNPA and NBAP exhibit a distinct absorption band near 2760 cm^−1^, originated from the stretched vibration of the -CH_3_ groups in 3-chloro-2-hydroxypropyltrimethylammonium chloride. A comparison between bentonite-contained samples (NBAP and NBNP) and those without bentonite (CNPA and NPA) revealed a distinct absorption band at 1037 cm^−1^ in the former, which corresponds to the asymmetric stretched vibration of the Si–O–Si bond in bentonite. The presence of these characteristic peaks confirms the successful synthesis of all target samples.

#### 2.1.2. SEM

**Figure 2:**
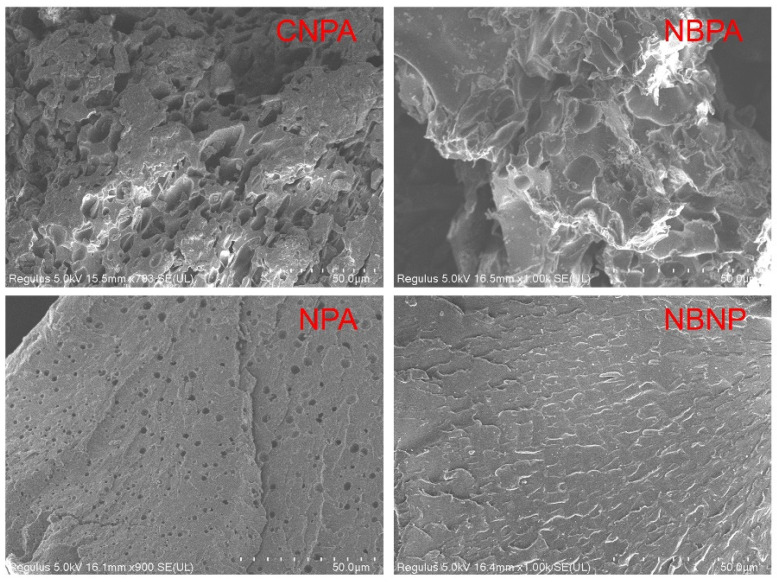
SEM characterization of the different samples.

Figure 2 presents a comparative analysis of the morphological characteristics of the samples obtained by scanned electron microscopy (SEM). Microstructural analysis revealed that the control samples without bentonite (the amphoteric polymer CNPA and the anionic polymer NPA) exhibited a porous surface morphology. Notably, CNPA displayed more pronounced pore dimensions, which may be attributed to the steric hindrance effects of the zwitterionic functional groups leading to a looser arrangement of molecular chains. Following the incorporation of bentonite, the surface porosity of both NBAP and NBNP samples was significantly reduced and virtually eliminated, indicating that the layered structure of bentonite effectively filled the defects within the polymer matrix. It is noteworthy that a comparison of the surface morphology between zwitterionic polymers (CNPA and NBAP) and anionic polymers (NPA and NBNP) revealed that the former exhibited more pronounced surface roughness and complex topological structures. This can be attributed to enhanced intermolecular interactions—such as electrostatic forces and hydrogen bonding—resulting from the zwitterionic functional groups, which led to a higher degree of phase separation; In contrast, the latter displayed relatively smoother surface characteristics, suggesting that the anionic functional groups promote a more homogeneous molecular distribution primarily through electrostatic repulsion.

### 2.2. Rheological Properties

**Figure 3:**
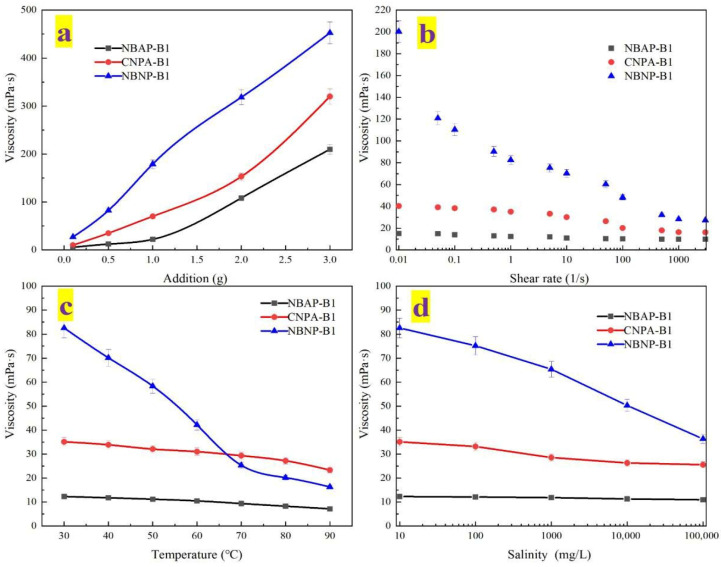
Rheological properties of different plugging systems: (**a**) polymer dosage, (**b**) shear rate, (**c**) temperature, and (**d**) salinity.

The effects of various conditions on the viscosity of the plugging systems were evaluated to analyze their rheological behavior. The results are presented in Figure 3. Figure 3a illustrates the relationship between polymer dosage and viscosity. The viscosity of all plugging systems increased with higher polymer loading. This behavior can be attributed to enhanced intermolecular entanglement resulting from the increased polymer chain concentration, which consequently elevated flow resistance. Moreover, the viscosity of NBAP-B1 was significantly lower than that of CNPA-B1 and NBNP-B1. This reduction can be explained by charge neutralization between the bentonite nanosheets and zwitterionic functional groups in NBAP-B1, which restricted the extension of polymer chains. In contrast, CNPA-B1 (without bentonite) and NBNP-B1 (where electrostatic repulsion dominates between anionic groups and bentonite) exhibited greater molecular chain mobility, resulting in higher viscosity. Figure 3b depicts the relationship between shear rate and viscosity. The viscosity of the systems decreased with increasing shear rate, indicating shear-thinning behavior. Furthermore, NBAP-B1 exhibited superior shear stability compared to CNPA-B1 and NBNP-B1. This enhanced performance is attributed to the dynamically reversible crosslinked network formed between the zwitterionic functional groups and bentonite nanosheets in NBAP-B1, which enables rapid structural reorganization under shear stress. In contrast, CNPA-B1 (lacking bentonite reinforcement) and NBNP-B1 (relying solely on anionic interactions) demonstrated weaker network recovery capabilities. Figure 3c,d illustrated the effects of temperature and salinity on the viscosity of the plugging systems, respectively. The viscosity gradually decreased with increasing temperature or salinity. Among them, NBNP-B1 exhibited the most pronounced viscosity reduction, while NBAP-B1 and CNPA-B1 showed relatively minor decreases. This difference can be attributed to the fact that the zwitterionic structures in both NBAP-B1 and CNPA-B1 mitigate the effects of temperature and salinity variations through intramolecular and intermolecular charge balance. In contrast, NBNP-B1 relies solely on electrostatic repulsion from anionic groups, making it more susceptible to thermal dissociation and charge shielding under high-salinity conditions.

### 2.3. Gelation Performance

#### 2.3.1. Gelation Time

**Figure 4:**
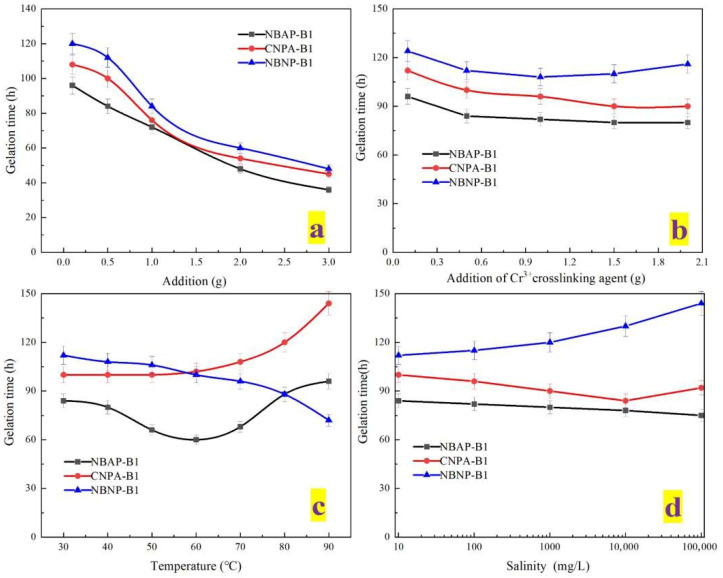
Gelation time of different plugging systems: (**a**) polymer dosage, (**b**) crosslinker concentration, (**c**) temperature, and (**d**) salinity.

The influence of various conditions on the gelation time of the plugging systems is presented in Figure 4. Figure 4a illustrates the relationship between polymer dosage and gelation time. As the polymer loading increased, the gelation time of the system gradually decreased. Among the different plugging systems, the gelation time followed the order: NBAP-B1 < CNPA-B1 < NBNP-B1. This behavior can be attributed to the synergistic effect between the zwitterionic groups (coexisting positive and negative charges) and bentonite in NBAP-B1, which accelerated the formation of a crosslinked network. In comparison, gelation in CNPA-B1 relied solely on the self-assembly of zwitterionic moieties, resulting in a moderately slower process. In contrast, NBNP-B1 exhibited the longest gelation time due to strong electrostatic repulsion among anionic groups, which imposed the greatest resistance to network formation. Figure 4b demonstrates the effect of Cr^3+^ crosslinker dosage on gelation time. As the concentration of Cr^3+^ crosslinker increased, the gelation time of NBAP-B1 and CNPA-B1 gradually decreased. In contrast, NBNP-B1 exhibited a non-monotonic trend, with gelation time initially decreasing and then increasing. This difference arises because the charge balance of zwitterionic groups in NBAP-B1 and CNPA-B1 enables uniform crosslinking with Cr^3+^ ions. In contrast, at high Cr^3+^ concentrations, the anionic groups in NBNP-B1 undergo excessive crosslinking, resulting in an overly dense network structure that restricts molecular chain mobility and consequently prolongs the gelation time. Figure 4c shows that as temperature increased, the gelation time of NBAP-B1 initially decreased and then increased, while that of CNPA-B1 rose gradually, and NBNP-B1 exhibited a consistent decrease. The non-monotonic change in gelation time for NBAP-B1 first decreasing and then increasing—can be explained by the synergistic effect between the zwitterionic groups and bentonite. Moderate heating promotes crosslinking, whereas excessively high temperatures disrupt the charge balance, ultimately delaying gel formation; The gradual increase in gelation time for CNPA-B1 is attributed to the insufficient thermal stability of the purely zwitterionic network. The consistent decrease in gelation time observed for NBNP-B1 is primarily driven by the thermally activated chelation between anionic groups and Cr^3+^ ions. Figure 4d shows that as salinity increased, the gelation time of NBAP-B1 decreased, while that of CNPA-B1 initially decreased and then increased. In contrast, NBNP-B1 exhibited a gradual increase in gelation time. The decrease in gelation time for NBAP-B1 is due to the compression of the electric double layer by salt ions, which enhances the crosslinking efficiency between zwitterionic groups and Cr^3+^; The gelation time of CNPA-B1 decreased initially and then increased with rising salinity. This non-monotonic trend can be explained by the fact that low salt concentrations promote chain expansion of the zwitterionic polymer, whereas high salt levels weaken charge-driven interactions, ultimately impairing crosslinking; The gelation time of NBNP-B1 increased consistently due to the suppression of crosslinking caused by charge shielding of anionic groups under high salinity. Furthermore, a comprehensive analysis of Figure 4 reveals that the gelation time of the plugging systems ranges from 20 to 150 h, indicating that it can be controllably adjusted to meet specific reservoir plugging requirements.

#### 2.3.2. Gelation Strength

**Figure 5:**
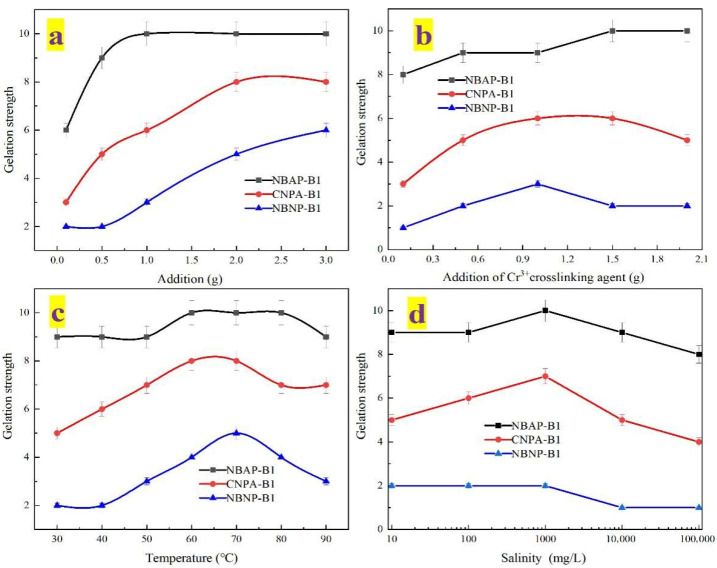
Gelation strength of different plugging systems: (**a**) polymer dosage, (**b**) crosslinker concentration, (**c**) temperature, and (**d**) salinity.

The influence of various conditions on the gelation strength of the plugging systems was investigated, and the corresponding results are presented in Figure 5. Figure 5a illustrates the relationship between polymer dosage and gelation strength. As the polymer concentration increased, the gelation strength of the system gradually enhanced. This enhancement can be attributed to the increased number of polymer chains, which provide more crosslinking sites and promote intermolecular entanglement, thereby facilitating the formation of a denser three-dimensional network structure and ultimately improving the gelation strength. Figure 4b illustrates the effect of Cr^3+^ crosslinker concentration on gelation strength. As the crosslinker dosage increased, the gelation strength of NBNP-B1 and CNPA-B1 initially rose and then declined, whereas that of NBAP-B1 increased initially and subsequently plateaued. This divergence occurs because excessive Cr^3+^ in NBNP-B1 and CNPA-B1 causes over-crosslinking, resulting in network embrittlement and consequent strength reduction. In contrast, the bentonite reinforcement and charge-balancing effect of zwitterionic groups in NBAP-B1 enable the maintenance of a stable crosslinked structure even at high Cr^3+^ concentrations. As shown in Figure 4c,d, the gelation strength of all systems initially increased and then decreased with rising temperature or salinity. This non-monotonic behavior occurs because moderate increases in temperature enhance molecular chain mobility, while higher salinity compresses the electric double layer—both of which improve crosslinking efficiency initially. However, beyond a critical point, elevated temperatures disrupt the network integrity, and excessive salinity induces charge shielding that weakens intermolecular interactions, ultimately leading to a decline in gelation strength. Furthermore, a comparison of gelation strength among the different systems revealed the following order: NBNP-B1 < CNPA-B1 < NBAP-B1. NBAP-B1 achieved the highest gelation strength, reaching grade J (the strongest level), whereas CNPA-B1 and NBNP-B1 attained maximum strengths of grade H and grade F, respectively. This difference is primarily due to the synergistic effect between zwitterionic groups and bentonite nanosheets in NBAP-B1, which facilitates the formation of the most dense and stable three-dimensional network. CNPA-B1 relies solely on zwitterionic self-assembly, resulting in a moderately effective yet suboptimal network structure. In contrast, the electrostatic repulsion between anionic groups and bentonite in NBNP-B1 leads to the weakest structural integrity among the three systems.

#### 2.3.3. Gel Microstructure

**Figure 6:**
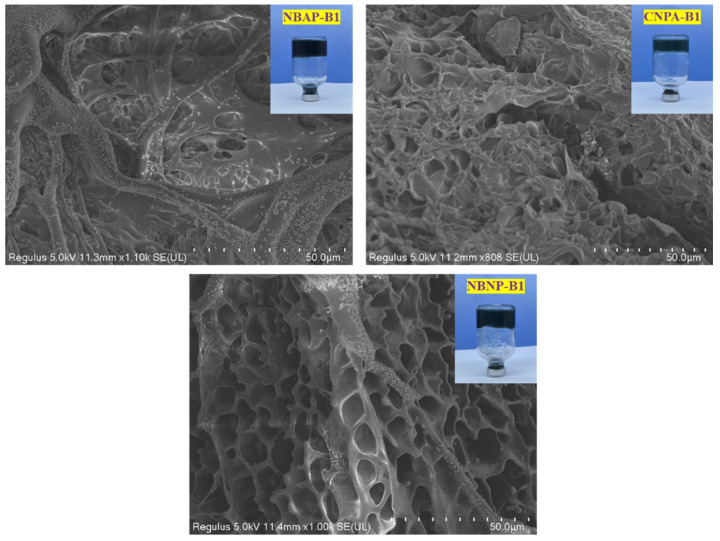
Microstructural images of gels from different plugging systems.

Figure 6 displays the microstructures of the gels formed by the different plugging systems. As shown in the image, the NBAP-B1 gel exhibits a uniform surface with a small number of fine pores, whereas the NBNP-B1 gel possesses a loose structure with larger voids. This enhanced structural integrity in NBAP-B1 arises from the strong electrostatic attraction between the cationic quaternary ammonium groups and the negatively charged surfaces of bentonite, combined with hydrogen bonding between the anionic sulfonate groups and the hydroxyl groups on bentonite. These synergistic interactions facilitate the formation of a dense and robust crosslinked network. In contrast, the loose structure of NBNP-B1 results primarily from electrostatic repulsion between the anionic groups and the negatively charged bentonite surfaces. It is particularly noteworthy that although CNPA-B1 contains no bentonite, its zwitterionic nature still enables the formation of a relatively uniform fine-network structure due to intramolecular self-assembly of oppositely charged groups. However, the absence of a nano-reinforcing phase leads to significantly inferior structural strength compared to NBAP-B1.

### 2.4. Injectability

**Figure 7:**
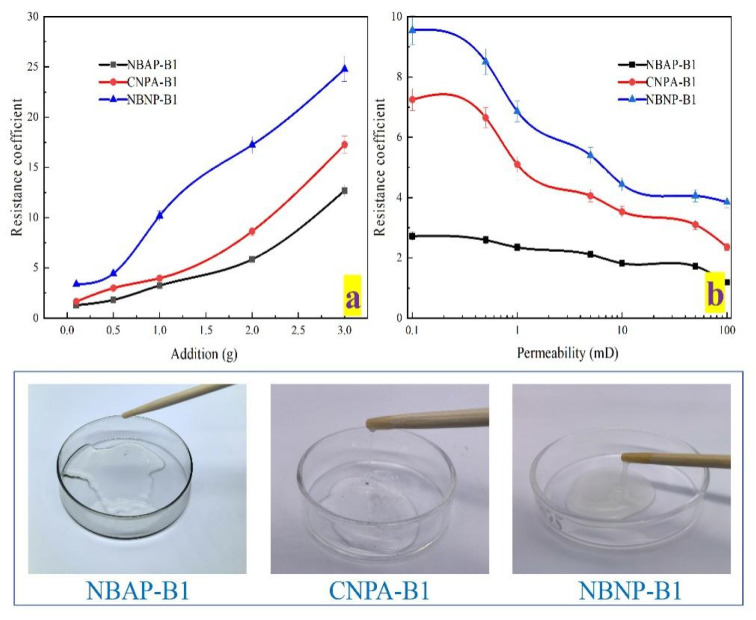
Resistance factor of different plugging systems: effects of polymer concentration (**a**) and permeability (**b**).

A higher resistance factor indicates poorer injectability. The effects of various conditions on the injectability of the plugging systems are presented in Figure 7. As shown in Figure 7a, the resistance factor of the plugging systems gradually increased with higher polymer loading. This increase can be attributed to enhanced intermolecular chain entanglement and a strengthened network structure resulting from higher polymer concentration, which collectively elevate the flow resistance of the plugging system. Meanwhile, Figure 7b demonstrates the influence of permeability on injectability. The resistance factor gradually decreased as permeability increased. This reduction occurs because higher permeability results in larger or better-connected pore channels within the reservoir, allowing polymer molecules to pass through more readily with reduced entrapment and clogging, thereby lowering the resistance factor. Furthermore, the resistance factors of the plugging systems followed the order: NBAP-B1 < CNPA-B1 < NBNP-B1. In NBAP-B1, the zwitterionic polymer chains exhibit electrostatic repulsion toward both the negatively charged nano-bentonite and the rock surfaces. This causes the polymer chains to adopt a more coiled conformation, reducing effective adsorption and bridging at pore throats, thereby resulting in the lowest flow resistance.

### 2.5. Plugging Performance

#### 2.5.1. Breakthrough Pressure

**Figure 8:**
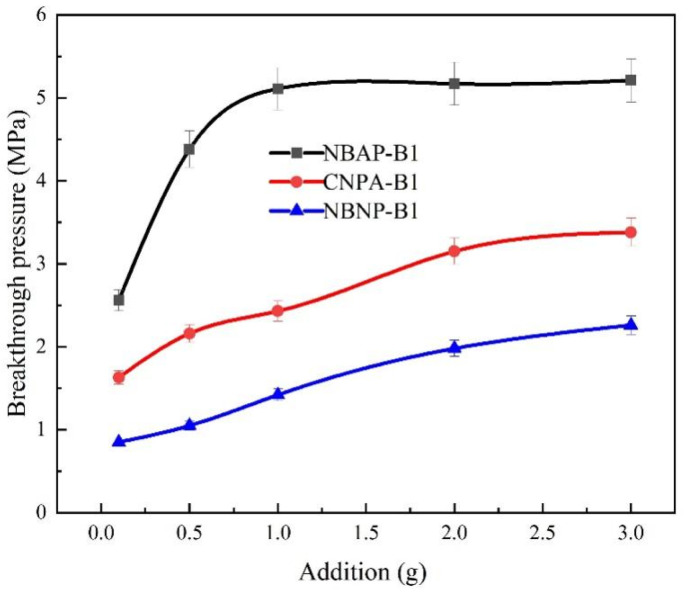
Breakthrough pressure of different plugging systems.

Figure 8 illustrates the relationship between polymer dosage and breakthrough pressure in the plugging systems. As the polymer concentration increased, the breakthrough pressure of the systems also rose. This increase can be attributed to the enhanced intermolecular entanglement and adsorption resulting from higher polymer concentration, which promotes the formation of a denser and more robust plugging network. Consequently, a higher pressure is required to penetrate this reinforced structure. Furthermore, the breakthrough pressure of the plugging systems followed the order: NBNP-B1 < CNPA-B1 < NBAP-B1. The superior performance of NBAP-B1 can be attributed to the combined effects of strong adsorption and complexation capabilities from its zwitterionic functional groups, as well as the physical filling and bridging actions provided by bentonite. These synergistic interactions enhance the strength and density of the gel network, resulting in the highest breakthrough pressure. This is because the anionic functional groups in NBNP-B1 experience electrostatic repulsion from both the negatively charged nano-bentonite and the rock surfaces, hindering the formation of an effective seal. As a result, NBNP-B1 exhibits the lowest breakthrough pressure; In contrast, the zwitterionic polymer in NBAP-B1 facilitates strong adsorption and bridging of nanoparticles via its cationic groups interacting with negatively charged surfaces, leading to the formation of a dense plugging barrier. This mechanism accounts for its highest breakthrough pressure.

#### 2.5.2. Plugging Efficiency

**Figure 9:**
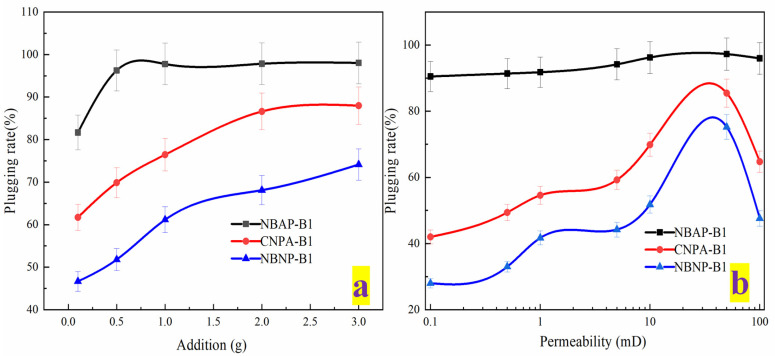
Plugging efficiency of different plugging systems: effects of polymer concentration (**a**) and permeability (**b**).

The influence of various conditions on the plugging efficiency of the plugging systems is presented in Figure 9. As shown in Figure 9a, the plugging efficiency gradually increased with higher polymer loading. This improvement can be attributed to enhanced intermolecular adsorption, bridging effects, and a denser network structure resulting from the increased polymer concentration, which collectively contribute to more effective pore throat blockage and consequently higher plugging efficiency. As observed in Figure 9b, the plugging efficiency of the systems initially increased and then decreased with rising core permeability. This trend occurs because in low-permeability cores, the narrow pore throats restrict the penetration of polymer molecules, limiting effective plugging. Conversely, in high-permeability cores, the larger pore throats make the plugging structure more susceptible to erosion and mechanical failure under flow conditions; Medium-permeability cores possess pore throat dimensions that best match the size of polymer chains/aggregates, thereby enabling optimal bridging and retention effects, which results in the highest plugging efficiency. Furthermore, the plugging efficiency of the different systems followed the order: NBNP-B1 < CNPA-B1 < NBAP-B1. The superior performance of NBAP-B1 is due to the strong adsorption and nanoparticle bridging facilitated by its cationic groups interacting with negatively charged surfaces, resulting in the densest and most stable plugging layer. In comparison, CNPA-B1 relies solely on the self-assembly and bridging of the zwitterionic polymer itself, leading to moderately effective but inferior plugging strength; In contrast, the anionic polymer in NBNP-B1 experiences electrostatic repulsion from the negatively charged components, hindering effective retention and plugging, thereby resulting in the lowest plugging efficiency.

### 2.6. Oil Displacement Performance

**Figure 10:**
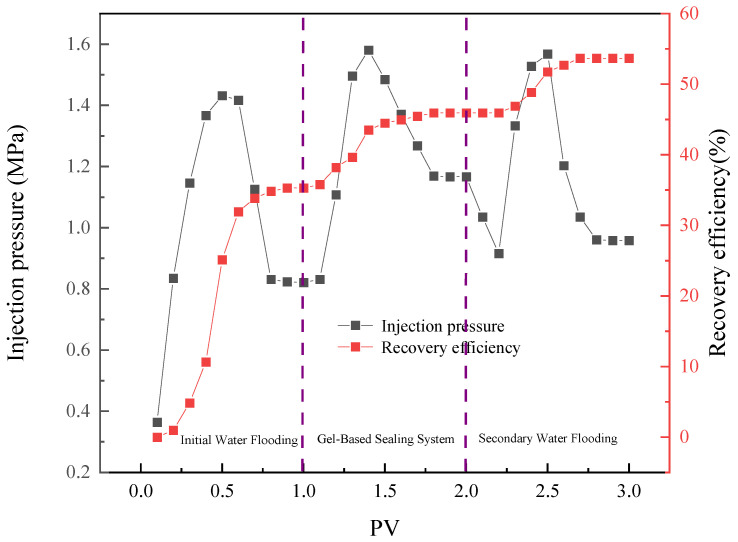
Oil Displacement efficiency of the plugging system NBAP-B1 at different stages.

As shown in Figure 10, the profile control and oil displacement process of the plugging system NBAP-B1 can be divided into three distinct stages. The first stage involves initial water flooding, during which the maximum injection pressure reached 1.43 MPa, resulting in an oil recovery rate of 35.3%. In the second stage, the NBAP-B1 system was injected and allowed to gel, leading to a rise in the maximum injection pressure to 1.58 MPa and an increase in the oil recovery rate to 45.94%. During the third stage, subsequent water flooding was conducted, with the maximum injection pressure stabilizing at 1.57 MPa and the recovery rate further rising to 53.67%. During the initial water flooding process, due to the heterogeneity of the core, injected water primarily flowed through the high-permeability zones (10 mD), effectively displacing crude oil from these regions. After the injection of the plugging system, the agent predominantly entered and blocked the high-permeability zones, while a small amount of the low-viscosity polymer penetrated into the low-permeability zones (5 mD). This resulted in an increase in injection pressure and an enhancement in oil recovery by 10.64%. Following gelation, subsequent water flooding was carried out, during which the plugging system mitigated core heterogeneity by diverting injected water into low-permeability zones. This led to a notable increase in injection pressure, effectively displacing trapped crude oil from these previously under-swept areas and thereby improving overall sweep efficiency. As a result, the cumulative enhancement in oil recovery reached 18.37%.

## 3. Conclusions

This study successfully developed a nanobentonite-reinforced amphoteric polyacrylamide composite gel (NBAP-B1), achieving three key innovations in the field of deep profile control and displacement materials through molecular structure design and nanocomposite technology:

First, we innovatively adopted a sequential reaction process to simultaneously incorporate anionic/cationic functional groups into the polymer backbone, constructing a unique dual-electronic molecular structure. This zwitterionic characteristic provides the material with exceptional salt tolerance and, through synergy with nanobentonite, forms a dense three-dimensional network unattainable in conventional polymers.

Second, we broke through the conventional approach of using nanomaterials merely as fillers. By combining chemical grafting with physical compounding, nanobentonite platelets were transformed into integral components of the polymer network. This design not only significantly enhances the mechanical strength of the gel but also endows the system with the unique property of “low initial viscosity-high strength evolution,” successfully resolving the technical challenge of balancing injectability and blocking performance during deep fluid diversion.

Most importantly, this research provides a precisely tunable solution for enhanced oil recovery in low-permeability reservoirs. The adjustable gelation time (20–150 h) and adaptive plugging characteristics demonstrated by the NBAP system enable customized design according to actual reservoir conditions. While achieving efficient fluid diversion, it enhances oil recovery by 18.37%, showing promising application prospects. These results indicate its promising potential for conformance control applications.

## 4. Materials and Methods

### 4.1. Materials

Acrylamide (AM, purity > 99%) was obtained from Tianjin Komiou Chemical Reagent Co., Ltd. (Tianjin, China); *N*,*N*′-Methylenebisacrylamide, ammonium persulfate, sodium hydroxide, and absolute ethanol (purity > 99.9%) were supplied by Tianjin Tianli Chemical Reagent Co., Ltd. (Tianjin, China).; Anhydrous chromium chloride, 3-chloro-2-hydroxypropyltrimethylammonium chloride, sodium 2-chloroethanesulfonate, thiourea, potassium bromide, and other related reagents (purity > 99.9%) were supplied by Shanghai Macklin Biochemical Technology Co., Ltd. (Shanghai, China); Polyvinyl alcohol (PVA) was obtained from Sigma-Aldrich (Shanghai, China)Trading Co., Ltd. (Shanghai, China) with a molecular weight ranging from 31,000 to 50,000 and a purity greater than 99.9%. All chemicals used were of analytical grade. Nanobentonite (purity > 96%, particle size: 200–500 nm) was obtained from Tianjin Yandong Haotian Mineral Products Co., Ltd. (Tianjin, China). The simulated oil used in this study was authentic crude oil sourced from the Chang 7 reservoir in Block Xi 233 of the Changqing Oilfield. It has a viscosity of 1.59 mPa·s and a density of 0.74 g/cm^3^ at 65 °C. The crude oil is predominantly composed of C5-C10 hydrocarbons, which account for approximately 80% of its composition. Hydrocarbons in the C11–C20 range constitute approximately 20% of the composition, while components above C20 are present in minor amounts. Notably, no components ≥ C29 were detected, consistent with classification as a light crude oil.

### 4.2. Synthesis of Bentonite/Amphoteric Polyacrylamide Composite Polymer NBAP

**Figure 11:**
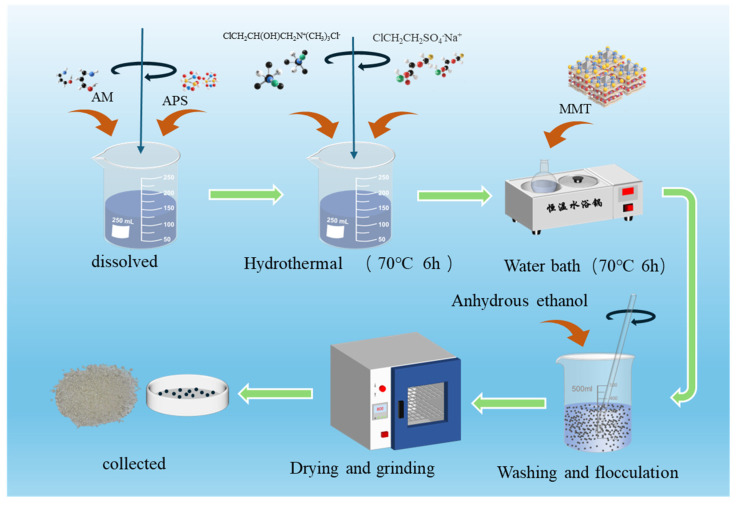
Schematic illustration of the polymer synthesis.

20 g of AM were dissolved in 80 g of deionized water under stirring. Subsequently, 0.2 g of ammonium persulfate was added to the solution, and the reaction was carried out at 65 °C under continuous stirring for 45 min. A total of 1 g of 3-chloro-2-hydroxypropyltrimethylammonium chloride and 2 g of sodium 2-chloroethanesulfonate were dissolved in 10 g of deionized water. The resulting solution was then added to the previously prepared mixture. The pH of the system was adjusted to 12 using a NaOH solution, and the temperature was raised to 70 °C. The reaction proceeded under stirring for 6 h to obtain the amphoteric polymer, denoted as CNPA. Subsequently, 0.2 g of nano-bentonite was added to the mixture, and the reaction was continued at 70 °C for an additional 6 h. The product was then purified via flocculation using a water-ethanol mixture, followed by repeated washing and filtration. After drying at 80 °C, the solid was ground into a fine powder to obtain the nano-bentonite/amphoteric polyacrylamide composite polymer, designated as NBAP. The synthetic route is illustrated in Figure 11. For comparison with NBAP, a nano-bentonite/anionic polyacrylamide composite (designated as NBNP) and a pure anionic polymer (denoted as NPA) were synthesized following the same procedure but without the addition of 3-chloro-2-hydroxypropyltrimethylammonium chloride.

### 4.3. Structural Characterization of NBAP

The functional groups of the NBAP composite were characterized using an INVENIO Fourier transform infrared (FTIR) spectrometer (Bruker Corporation, Bremen, Germany) to verify the successful synthesis of the polymer. Potassium bromide (KBr) was dried using a heating lamp and subsequently ground in a mortar. The sample was then added at a mass ratio of 1:100 (sample to KBr) for further homogenization. The mixture was thoroughly ground with a pestle to achieve a uniform particle size of less than 2.5 μm and then pressed into pellets using a hydraulic press; The resulting pellet was finally transferred to the FTIR spectrometer for scanning. Data were acquired and analyzed using dedicated software to identify characteristic peaks corresponding to various functional groups. The scanning was performed over a wavenumber range of 4400 to 400 cm^−1^.

The microstructure of the synthesized NBAP sample was characterized using a Regulus8100 field-emission scanning electron microscope (FE-SEM; Hitachi, Tokyo, Japan). Specifically, the sample was mounted on a conductive stage, freeze-fractured in liquid nitrogen, and sputter-coated with a thin layer of gold. The prepared specimen was then transferred to the FE-SEM chamber for observation to analyze the influence of nanoparticles on the polymeric morphology.

### 4.4. Formulation of a Nanopolymer-Based Plugging Agent System

NBAP-B1 and polyvinyl alcohol (PVA) were first dissolved separately in deionized water under stirring. The two solutions were then combined, followed by the addition of a pre-prepared Cr^3+^ crosslinking agent, ammonium persulfate solution, and the oxygen scavenger thiourea. After thorough mixing, the resulting solution was transferred into glass vials, which were sealed and placed in an oven. Gelation behavior was monitored at specific time intervals under controlled temperature conditions. The composition of each formulation is summarized in Table 1. The Cr^3+^ crosslinking agent was prepared in the laboratory according to the following specific formulation: Add 1 g [Cr(H_2_O)_6_]Cl_3_ and 1.95 g CH_3_COOH to 100 mL of water solution, added NaOH to adjust the pH to above 12, passed nitrogen for 30 min, reacted at 50 °C for 2 h, and storeed it in a sealed container at room temperature for 24 h. The amphoteric polymer plugging system CNPA-B1 and the bentonite/anionic polymer plugging system NBNP-B1 were prepared following the identical procedure.

### 4.5. Rheological Property Evaluation

This study investigated the adaptability of plugging systems under various conditions typical of low-permeability reservoirs. Following the experimental procedure outlined in Section 4.4, three plugging systems with different compositions—namely NBAP-B1, CNPA-B1, and NBNP-B1—were prepared. The rheological properties of each system were systematically characterized using an NDJ-5S rotational viscometer (Brookfield Engineering Laboratories, Inc., USA). The effects of polymer type (NBAP, CNPA, or NBNP), dosage (0.1 g, 0.5 g, 1 g, 2 g, or 3 g), temperature (30~90 °C), and shear rate (0.01~3000 s^−1^) on the viscosity of the plugging systems were systematically investigated. Additionally, simulated formation water with varying salinities was prepared by dissolving sodium chloride in deionized water. The plugging systems were formulated using this brine instead of pure deionized water to evaluate the effect of salinity (100 mg/L, 1000 mg/L, 10,000 mg/L, and 100,000 mg/L) on their viscosity. It is noteworthy that when investigating the influence of a specific factor, all other parameters were held constant. Specifically, the polymer dosage, temperature, shear rate, and salinity were fixed at 0.5 g, 30 °C, 1 s^−1^, and 0 mg/L, respectively.

### 4.6. Gelation Performance Evaluation

This study investigated the gelation time and gelation strength of various plugging systems to comprehensively evaluate their gelation performance. Following the experimental procedure described in Section 4.4, plugging systems with different compositions (NBAP-B1, CNPA-B1, and NBNP-B1) were prepared. Each system was transferred into glass vials, which were then placed at different temperatures. The gelation time and gelation strength were monitored and recorded accordingly. The gelation strength was characterized using the Marathon gelation strength Codes method. To facilitate comparison of strength variations among different systems, the gelation levels were quantified numerically, as detailed in Table 2. The effects of polymer type (NBAP, CNPA, or NBNP), polymer dosage (0.1, 0.5, 1, 2, or 3 g), Cr^3+^ crosslinker volume (0.1, 0.5, 1, 2, or 3 mL), and temperature (30–90 °C) on the gelation performance of the plugging systems were systematically investigated. Additionally, simulated formation water with varying salinities was prepared by dissolving sodium chloride in deionized water. The plugging systems were formulated using this brine instead of pure deionized water to evaluate the influence of salinity—specifically at concentrations of 100, 1000, 10,000, and 100,000 mg/L—on the gelation performance. It should be noted that when evaluating the effect of a specific variable, all other parameters were maintained constant. The baseline conditions were set as follows: polymer dosage = 0.5 g, temperature = 80 °C, and salinity = 0 mg/L. Furthermore, the microstructure of the gel formed under these conditions was examined using scanning electron microscopy (SEM).

### 4.7. Injectability Testing

**Figure 12:**
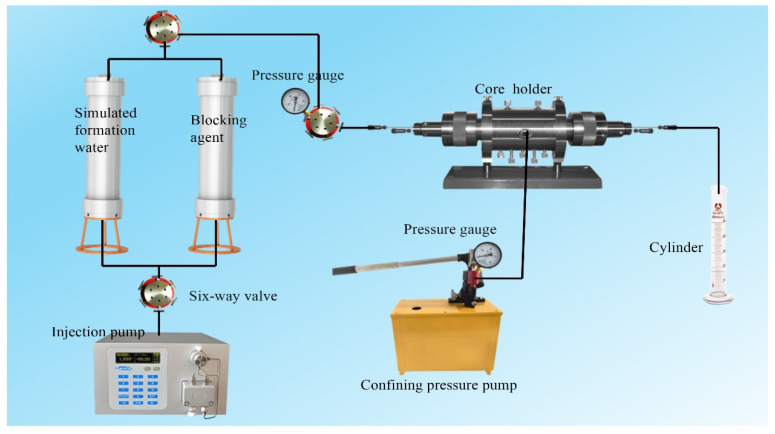
Schematic diagram of the fluid flooding apparatus.

The injectability of various plugging systems was evaluated by measuring the resistance factor to comprehensively assess their injection performance. Following the procedure outlined in Section 4.4, systems with different compositions (NBAP-B1, CNPA-B1, and NBNP-B1) were prepared. The influence of polymer dosage (NBAP, CNPA, and NBNP) on injectability was systematically investigated. Fifteen core samples, each with a length of 8.0 cm, diameter of 3.8 cm, and permeability of 10 mD, were selected for the experiment. The core samples were dried and subsequently loaded into a core holder. The flow apparatus was then assembled as illustrated in Figure 12. Deionized water was injected into the core at a flow rate of 0.1 mL/min until a stable pressure was attained, and the pressure difference (Δ*P_w_*) was recorded. Subsequently, the plugging system was injected at the same flow rate until pressure stabilization, and the corresponding pressure difference (Δ*P_b_*) was measured. The resistance factor for each system was calculated according to Equation (1).

(1) $$F_{r}=\frac{△P_{b}}{△P_{W}}\cdot \frac{Q_{W}}{Q_{b}}\cdot$$

The resistance factor F*_r_* is dimensionless. Δ*P_w_* and Δ*P_b_* represent the pressure differences (in MPa) during the injection of water and the plugging system, respectively. Similarly, *Q_w_* and *Q_b_* denote the flow rates (in mL/min) of water and the plugging system, respectively.

Additionally, plugging systems with a fixed polymer dosage of 0.5 g were prepared. Core samples of identical dimensions but varying permeabilities (0.1, 0.5, 1, 5, 10, 50, and 100 mD) were selected to evaluate the influence of permeability on the injectability of the plugging systems using the same experimental procedure.

### 4.8. Plugging Performance of NBAP-B1

The plugging performance of various systems was evaluated based on breakthrough pressure and plugging efficiency. Following the procedure described in Section 4.4, systems with different compositions (NBAP-B1, CNPA-B1, and NBNP-B1) were prepared. The effect of polymer dosage (NBAP, CNPA, and NBNP) on their injectability was also systematically investigated. Fifteen core samples, each measuring 8.0 cm in length and 3.8 cm in diameter with a uniform permeability of 10 mD, were selected for the experiment. After drying, the cores were loaded into a core holder. The flow apparatus was then connected according to the schematic shown in Figure 12. Deionized water was injected into the core at a constant flow rate of 0.1 mL/min until pressure stabilization was achieved. The injection pressure was recorded, and the initial permeability (K_a_) was calculated; The plugging system was then injected at the same flow rate until a stable pressure was achieved. Following the injection, the core was transferred to an oven and maintained at 80 °C under static conditions to facilitate gel formation; Finally, deionized water was injected again at the same flow rate to measure the post-treatment permeability K_b_. The plugging efficiency for each system was then calculated using Equation (2). Additionally, during the post-blocking water flooding process, the pressure at the inlet was recorded at the moment when the first water droplet appeared at the outlet. This pressure value was defined as the breakthrough pressure P_b_.

(2) $$\eta =\frac{K_{a}-K_{b}}{K_{a}}\times 100\%\cdot$$

η represents the plugging efficiency (%), while K_a_ and K_b_ denote the permeability before and after plugging (in mD), respectively. Additionally, plugging systems with a fixed polymer dosage of 0.5 g were prepared. Core samples with identical dimensions but varying permeabilities (0.1, 0.5, 1, 5, 10, 50, and 100 mD) were selected to evaluate the influence of permeability on the plugging efficiency using the same experimental procedure.

### 4.9. Flow and Oil Displacement Experiments

In the core flooding experiments, the change in oil displacement efficiency before and after the application of the plugging system was compared to evaluate its profile control and oil displacement performance. The NBAP-B1 system, which demonstrated the best plugging performance, was selected for this study with a consistent polymer concentration of 0.5 g. Oil displacement performance was analyzed following NBAP-B1 treatment. A heterogeneous artificial core was used, consisting of a high-permeability zone (10 mD) and a low-permeability zone (5 mD). Detailed core properties are provided in Table 3. The core was dried at 80 °C and then split along the center using a cutting machine to create an artificial fracture. The fracture was subsequently filled with sand and cemented. Following this, the core was saturated with oil and water to establish the initial oil saturation. First, vacuum the washed and dried core and apply pressure to saturate the formation water, making it reach a 100% water content state. Then, using simulated oil, slowly displace the water in the core at the reservoir temperature until no water is left. At this point, the initial water saturation and initial oil saturation of the core can be calculated by measuring the displaced water volume or by weighing. Finally, the core was mounted into a core holder, and the flow apparatus was connected according to the configuration illustrated in Figure 12. Deionized water was injected into the core at a flow rate of 0.1 mL/min until no more oil was produced at the outlet. Subsequently, the plugging system was injected at the same flow rate until the pressure stabilized. The core was then placed in an oven at 80 °C for static gelation; Finally, deionized water was injected again at the same flow rate until no additional oil was produced. The plugging efficiency of each system was then calculated using Equation (2). The brine used was NaCl-based simulated formation water with a salinity of 50,000 mg/L. The injection pressure and oil production were recorded throughout each stage of the process to evaluate the oil displacement performance of the different plugging systems.

## Figures and Tables

**Table 1:** Dosage of Individual Components in the Plugging System.

Number	Water (g)	One of the Following: NBAP, CNPA, or NBNP. (g)	PVA (g)	Cr^3+^ Crosslinking Agent (mL)	10% Ammonium Persulfate Solution (g)	Thiourea (g)
1	100	0.1	0.5	0.5	0.2	0.02
2	100	0.5	0.5	0.5	0.2	0.02
3	100	1	0.5	0.5	0.2	0.02
4	100	2	0.5	0.5	0.2	0.02
5	100	2.5	0.5	0.5	0.2	0.02

**Table 2:** Strength grades and corresponding numerical codes [43].

Strength Grade	A	B	C	D	E	F	G	H	I	J
Numbers	1	2	3	4	5	6	7	8	9	10

**Table 3:** Core properties of oil displacement experiments.

Core Number	Diameter (cm)	Length (cm)	Permeability (mD)	Porosity (%)	Initial Oil Saturation (%)
LP-1	3.801	8.006	5/10	19.45	58.55

## Data Availability

The original contributions presented in this study are included in the article. Further inquiries can be directed to the corresponding authors.

## Abbreviations

The following abbreviations are used in this manuscript:

FTIR	Fourier transform infrared spectroscopy
SEM	scanning electron microscopy
NBAP	nano bentonite/amphoteric polyacrylamide
CNAP	Amphoteric polymer
NBNP	Nano bentonite/anionic polyacrylamide
NPA	Anionic polymer
PV	Pore volume
